# The Chemical and Pharmacological Research Progress on a Kind of Chinese Herbal Medicine, *Fructus Malvae*

**DOI:** 10.3390/molecules27175678

**Published:** 2022-09-02

**Authors:** Xiaoyu Li, Xianglei Wang, Menglu Zhao, He Zhang, Chao Liu

**Affiliations:** 1 Nanjing University of Chinese Medicine, Nanjing 210023, Jiangsu, China; 2 Department of Pharmaceutical Analysis, Affiliated Hospital of Integrated Traditional Chinese and Western Medicine, Nanjing University of Chinese Medicine, Nanjing 210028, Jiangsu, China; 3 Department of Endocrinology, Affiliated Hospital of Integrated Traditional Chinese and Western Medicine, Nanjing University of Chinese Medicine, Nanjing 210028, Jiangsu, China; 4 Pharmaceutical Sciences, Institute of Medical, Pharmaceutical and Health Sciences, Kanazawa University, Kanazawa 920-1192, Ishikawa, Japan; 5 Department of Pharmacy, The Affiliated Dongnan Hospital of Xiamen University, Zhangzhou 363000, Fujian, China

**Keywords:** Chinese herbal medicine, *Fructus Malvae*, chemical constituents, pharmacological activity, anti-diabetes, anti-tumor, nortangeretin-8-O-β-d-glucuronopyranoside

## Abstract

Since the outbreak of the COVID-19 pandemic, traditional Chinese medicine has played an important role in the treatment process. Furthermore, the discovery of artemisinin in *Artemisia annua* has reduced the incidence of malaria all over the world. Therefore, it is becoming urgent and important to establish a novel method of conducting systematic research on Chinese herbal medicine, improving the medicinal utilization value of traditional Chinese medicine and bringing great benefits to human health all over the world. *Fructus Malvae*, a kind of Chinese herbal medicine which has been recorded in the “*Chinese Pharmacopoeia*” (2020 edition), refers to the dry, ripe fruits of *Malva verticillata* L. Recently, some studies have shown that *Fructus Malvae* exhibits some special pharmacological activities; for example, it has diuretic, anti-diabetes, antioxidant and anti-tumor properties, and it alleviates hair loss. Furthermore, according to the reports, the active ingredients separated and identified from *Fructus Malvae* contain some very novel compounds such as nortangeretin-8-O-β-d-glucuronopyranoside and 1-O-(6-deoxy-6-sulfo)-glucopyranosyl-2-O-linolenoyl-3-O-palmitoyl glyceride, which could be screened as important candidate compounds for diabetes- or tumor-treatment drugs, respectively. Therefore, in this research, we take *Fructus Malvae* as an example and systematically summarize the chemical constituents and pharmacological activity research progress of it. This review will be helpful in promoting the development and application of *Fructus Malvae* and will also provide an example for other investigations of traditional Chinese medicine.

## 1. Introduction

Since the outbreak of the COVID-19 pandemic, the economic development and public health services in many countries and regions have been influenced and challenged [1]. Traditional Chinese medicine (TCM), with thousands of years of history of use and practice in China, has played an important role in the treatment of diseases and the maintenance of human health. In the fight against the COVID-19 pandemic in China, traditional Chinese medicine (TCM) is widely used and has achieved remarkable success [2]. Additionally, the discovery of artemisinin in *Artemisia annua* has reduced the incidence of malaria around the world [3]. Therefore, it is becoming urgent and important to establish a novel method of conducting systematic research on Chinese herbal medicine, improving the medicinal value of traditional Chinese medicine and bringing great benefits to human health all over the world.

**Figure 1:**
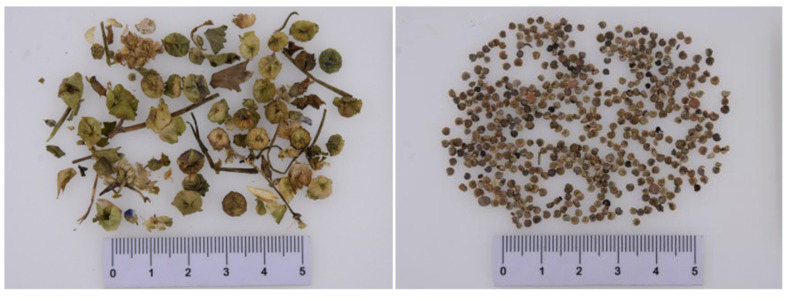
Pictures of *Fructus Malvae*.

*Fructus Malvae*, a kind of Mongolian medicinal material which has been included in “*Chinese Pharmacopoeia*” (2020 edition), refers to the dried and ripe fruit of *Malva verticillata* L. [4] (Figure 1). It is cold in nature, and sweet and astringent in taste, with the effects of clearing away heat, inducing diuresis and reducing swelling [4]. In a clinical setting, it is generally utilized for the treatment of urinary retention, edema, thirst, urinary tract infection, etc. The original production area of *Fructus Malvae* is Inner Mongolia [5]. Additionally, it also grows in Shandong, Hebei, Sichuan and many other provinces in China [6,7]. *Fructus Malvae* has been used for more than 2000 years in China. From “*Shen Nong’s Materia Medica*” to the “*Compendium of Materia Medica*” to the “*Illustrated Catalogue of Plants*” [8,9,10], there are plenty of related records and descriptions of it. Furthermore, *Fructus Malvae* is also included in four current editions of Chinese herbal medicine processing specifications, including the *Chinese Herbal Medicine Processing Specifications of Jiangxi Province* (2008 edition), the *Chinese Herbal Medicine Processing Specifications of Hunan Province* (2010 edition), the *Chinese Herbal Medicine Processing Specifications of Guangxi Province* (2007 edition) and the Chinese herbal medicine processing specification of Gansu Province (2009 edition). *Fructus Malvae* is included in the national medical insurance reimbursement list, as well. All of this indicates that it is very necessary and significant to further study the medicinal value of *Fructus Malvae*.

Recently, some studies have found that *Fructus Malvae* performs some very special pharmacological activities; for example, it has anti-diabetes, antioxidant and anti-tumor properties, it alleviates hair loss, etc. Furthermore, according to the reports, the active ingredients separated and identified from *Fructus Malvae* contain some very novel compounds, such as nortangeretin-8-O-β-d-glucuronopyranoside and 1-O-(6-deoxy-6-sulfo)-glucopyranosyl-2-O-linolenoyl-3-O-palmitoyl glyceride, which could be screened as important candidate compounds for diabetes- and tumor-treatment drugs, respectively. Myristoleic acid was isolated and identified from dichloromethane extract, and linolenic acid was isolated and identified from n-butanol extract, which are the two main active compounds of *Fructus Malvae* in the treatment of hair loss. Therefore, in this study, we take *Fructus Malvae* as an example and systematically summarize the chemical constituents and pharmacological activities of it. This review will be helpful in promoting the development and application of the medicinal value of *Fructus Malvae* and will also provide an example for the investigation of other traditional Chinese medicines.

## 2. Research Progress on Chemical Constituents

As a kind of Chinese herbal medicine with more than 2000 years of history of application, certain studies have been performed on the chemical constituents of *Fructus Malvae*. The medicinal parts studied include the fruit [7,11,12,13,14,15,16,17,18,19,20], the seed [21,22,23,24,25,26,27,28,29,30] and the stem, leaf and seed mixture [31,32,33,34,35] of *Malva verticillata* L. Common extraction methods include ultrasonic extraction [22,31], soaking in solution at room temperature [32,33,34], boiling in hot water [23] and refluxing extraction [4]. Solvents including 80% methanol [32,33,34], 90% ethanol [31], 95% ethanol [21], ethanol [22,24], distilled water [22,23], ethyl acetate [22], n-butanol [32], n-hexane [21] and methylene chloride [22] are often used for their extraction, elution and purification. The extraction, separation and purification process of the chemical components of *Fructus Malvae* is usually as follows: *Fructus Malvae* or the stem, leaf and seed mixture of *Malva verticillata* L. are extracted using a certain solvent through ultrasound, soaking at room temperature or heating under reflux. The extraction solution is filtered and concentrated under reduced pressure to obtain the corresponding extracts. Then, the extracts are dissolved and sequentially extracted using different solvents [32,33,34]. The compounds are separated and purified from different solvent fractions via column chromatography. Finally, the compounds are identified and characterized by using NMR, IR, FAB-MS, GC-MS, HPLC, UPLC-QTOF-MS or other instrumental analysis methods [32,33,34].

The chemical compounds identified from *Fructus Malvae* include 9 acid compounds (compounds **1**–**9**), 21 flavonoids (compounds **10**–**30**), 3 sterols (compounds **31**–**33**), 17 glycerides (compounds **34**–**50**), 24 volatile oils (compounds **51**–**74**), 9 polysaccharides (compounds **75**–**83**), 15 amino acids (compounds **84**–**98**) and 5 other compounds (compounds **99**–**103**). Among them, caffeic acid and ferulic acid are two phenolic acid compounds identified from *Fructus Malvae* [7,11,12,13,14,15,16,17]. The two compounds are often selected as indicator components for the qualitative or quantitative analysis of *Fructus Malvae* in the associated drug standards or literature. UPLC-QTOF-MS/MS semi-quantitative analysis shows that the contents of the flavonoids hypericin and kaempferol-3-O-rutinoside are the highest in the seeds of *Malva verticillata* L. [31]. In addition, Nortangeretin-8-O-β-d-glucuronide, isoscutellarein 8-O-glucuronopyranosid, hypolaetin 8-O-glucuronopyranoside, herbacetin 8-O-glucuronopyranoside, herbacetin 3-O-glucopyranosyl-8-O-glucuronopyranoside and isoscutellarein 7-O-glucopyranoside are six flavonoids identified from the stem, leaf and seed mixture of *Malva verticillata* L., and 8-O-glucuronide, attached to the flavonoid moiety, has rarely been reported in plant systems [32]. There are 13 glycosylglycerides included in 17 glycerides, according to the reports [33,34]. Among them, 1-O-galactopyranosyl-3-O-isostearoyl glyceride (compound **42**) is a novel glycosylglyceride compound. 1-O-(6-deoxy-6-sulfo)-glucopyranosyl-2-O-linolenoyl-3-O-palmitoyl glyceride and 1-O-(6-deoxy-6-sulfo)-glucopyranosyl-2,3-di-O-linolenoyl glyceride (compounds **38**, **39**) are two sulfoquinovosyl glycerides, which contain a unique chemical structure “(6-deoxy-6-sulfo)-a-d-glucopyranose” rarely reported in plants [34].

### 2.1. Acid Compounds

**Figure 2:**
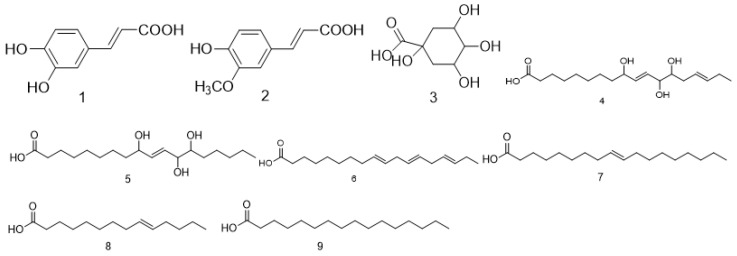
The chemical structures of acid compounds identified from *Fructus Malvae*.

For the acid compounds identified from *Fructus Malvae*, caffeic acid [7,11,12,13,14,15] and ferulic acid [7,15,16,17] are two phenolic acids and they are often screened as marker compounds of *Fructus Malvae* for qualitative or quantitative analysis in the associated drug standards or literature. In terms of extraction methods, in the *Chinese Pharmacopoeia* (2020 vision), *Fructus Malvae* was extracted using 70% ethanol under heating and refluxing, and then, caffeic acid was determined via thin-layer chromatography [4]. In addition, in some other studies, *Fructus Malvae* was also extracted via water decoction, ultrasonic extraction or Soxhlet extraction; then, the caffeic acid was determined via high-performance liquid chromatography [15]. The extraction, separation and identification methods of ferulic acid are similar to that of caffeic acid [15,16,17]. The stem, leaf and seed mixture of *Malva verticillata* L. was extracted using 90% ethanol under ultrasound; after that, four fatty-acid compounds (compounds **3**–**6**) were identified from extracts based on UPLC-QTOF-MS/MS analysis [31]. In another study, *Fructus Malvae* was extracted using ethanol, and then, the extraction was successively extracted using dichloromethane, ethyl acetate, water, n-hexane and other solvents. Finally, linolenic acid and oleic acid were isolated and identified from n-butanol extract [21] and myristoleic acid (compound **8**) was separated and identified from dichloromethane extract [22], which are three polyunsaturated fatty-acid compounds contained in *Fructus Malvae*. Additionally, palmitic acid, a kind of saturated fatty acid, was also identified using GC/MS from the water extract of *Fructus Malvae* [23]. The information and chemical structures of the acid compounds identified from *Fructus Malvae* are shown in Table 1 and Figure 2.

### 2.2. Flavonoids

**Figure 3:**
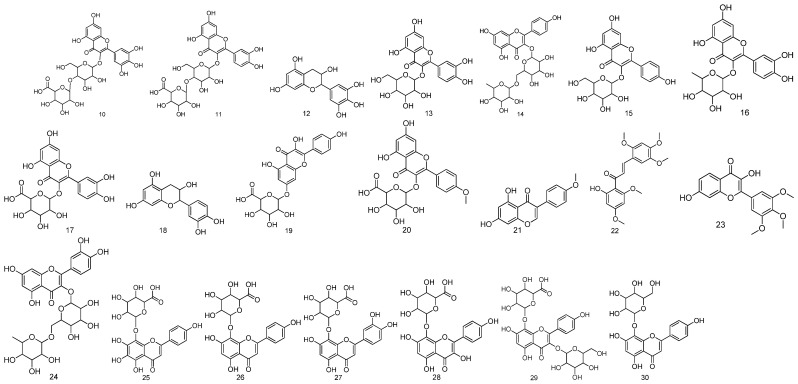
The chemical structures of flavonoids identified from *Fructus Malvae*.

For the flavonoids identified from *Fructus Malvae*, 21 flavonoids (compounds **10**–**30**) have been determined so far. A total of 14 flavonoid compounds (compounds **10**–**23**) were identified via UPLC-QTOF-MS/MS from a 90% ethanol extract of the stem, leaf and seed mixture of *Fructus Malvae* [31]. In another study, the stem, leaf and seed mixture was extracted using 80% methanol at room temperature for 24 h, and then, successively extracted using water, ethyl acetate and n-butanol. A total of six flavonoid compounds, including nortangeretin-8-O-β-d-glucuronopyranoside (compound **25**), isoscutellarein 8-O-β-d-glucuronopyranoside (compound **26**), hypolaetin8-O-β-d-glucuronopyranoside (compound **27**), herbacetin-8-O-β-d-glucuronopyranoside (compound **29**) and isoscutellarein 7-O-β-d-glucopyranoside (compound **30**), were separated and identified from the water extract [32]. Among the six flavonoids, ortangeretin-8-O-β-d-glucuronopyranoside is a new compound. The 5,6,7,8-tetrahydroxy group and the 8-O-glucuronide attached to the A ring of the flavonoid moiety are rarely reported in plants [32]. Additionally, the study also proved that 8-O-glucuronide attached to the flavonoid moiety was crucial for the antioxidant activity of *Fructus Malvae* [32]. The flavonoid rutin was separated and identified from the EtOAc extract of *Fructus Malvae* using UV, IR, MS and some other technologies [18]. The chemical names, molecular formulas and chemical structures of all the identified flavonoids are shown in Table 2 and Figure 3.

### 2.3. Sterols

**Figure 4:**
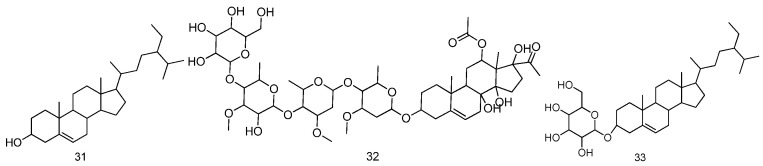
The chemical structures of sterol compounds identified from *Fructus Malvae*.

For sterols separated from *Fructus Malvae*, there are only three sterol compounds identified from *Fructus Malvae*. They are β-sitosterol, verticilloside and daucosterol. *Fructus Malvae* was ultrasonically extracted using ethanol for 6 h, and then, the extraction was successively extracted using dichloromethane, ethyl acetate and water. Sterols including β-sitosterol, verticilloside and daucosterol were identified from the dichloromethane extraction [22]. The above sterol compounds could also be separated using other extraction methods [18,23,24]. The information on and chemical structures of the sterol compounds identified from *Fructus Malvae* are shown in Table 3 and Figure 4.

### 2.4. Glycerides

**Figure 5:**
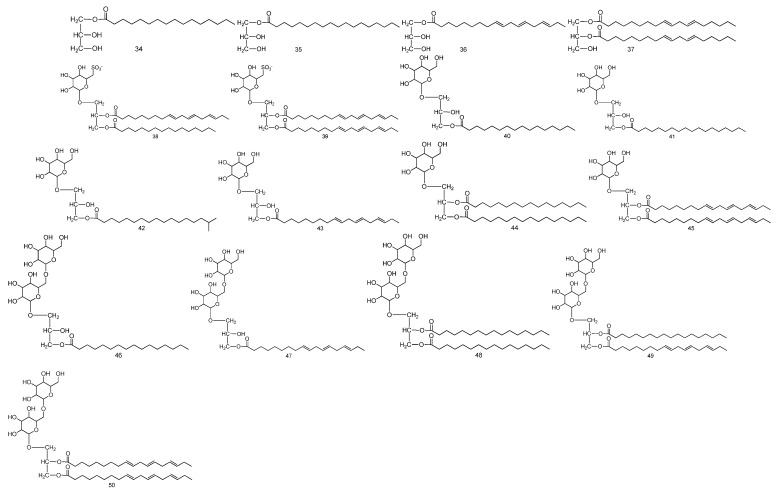
The chemical structures of glycerides identified from *Fructus Malvae*.

For glycerides, so far, there have been 17 glyceride compounds separated and identified from *Fructus Malvae* [33,34]. The stem, leaf and seed mixture of *Fructus Malvae* was extracted using 80% methanol; after that, the extract was dissolved and extracted using water, ethyl acetate and n-butanol, in sequence. After column chromatography, 17 glyceride compounds, including 13 glycosylglycerides, were identified from the n-butanol extraction through NMR, IR, FAB-MS and GC-MS analysis [33,34]. 1-O-palmitoyl glyceride, 1-O-stearoyl glyceride and 1-O-linolenoyl glyceride were three monoacylglyceride compounds, while 1,2-di-O-linoleoyl glyceride was a diacylglyceride compound. The above four glyceride compounds all showed antitumor activity [33]. Regarding the structure–activity relationship, monoacylglycerides have stronger antitumor activity than diacylglycerides. Furthermore, for monoacylglycerides, the longer the carbon chain of fatty acids, the better the antitumor activity; unsaturated fatty acids show better activity than saturated fatty acids [33]. Among the 13 glycosylglycerides, 1-O-galactopyranosyl-3-O-isostearoyl glyceride (compound **42**) is a novel glycosylglyceride compound. 1-O-(6-deoxy-6-sulfo)-glucopyranosyl-2-O-linolenoyl-3-O-palmitoyl glyceride and 1-O-(6-deoxy-6-sulfo)-glucopyranosyl-2,3-di-O-linolenoyl glyceride (compounds **38**, **39**) are two sulfoquinovosyl glycerides, and both of them contain a unique chemical structure “(6-deoxy-6-sulfo)-a-d-glucopyranose” which is rarely reported in plants [34]. The chemical names, molecular formulas and chemical structures of the 17 glycerides are shown in Table 4 and Figure 5.

### 2.5. Volatile Oils

**Figure 6:**
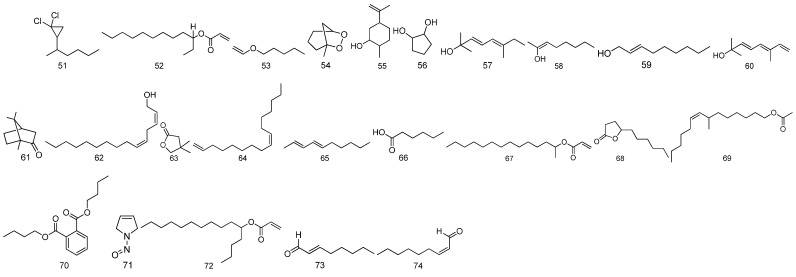
The chemical structures of volatile oils identified from *Fructus Malvae*.

For the volatile oils, there are 24 volatile oil compounds identified from *Fructus Malvae* in total. Volatile oils are often extracted via water-vapor distillation. An appropriate amount of *Fructus Malvae* powder is weighed and soaked in water for 12 h, and finally, a volatile oil extractor is used for steam distillation for 8 h. The effluent is collected and extracted using n-hexane; after that, it is dried using anhydrous sodium sulfate [19,20]. The information and chemical structures of the 24 volatile oil compounds, identified via GC-MS analysis, are shown in Table 5 and Figure 6.

### 2.6. Polysaccharides

**Figure 7:**
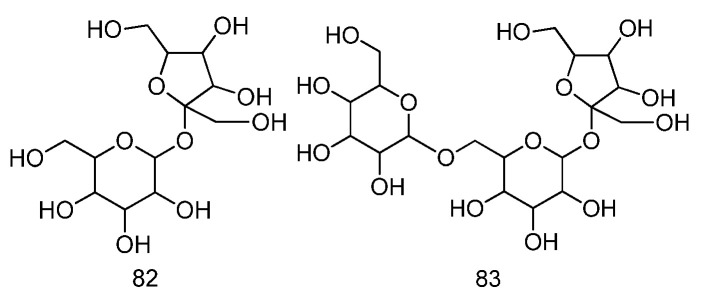
The chemical structures of polysaccharides identified from *Fructus Malvae*.

For the polysaccharides, seven polysaccharides and two oligosaccharides have been identified from *Fructus Malvae* so far. The water extraction and alcohol precipitation methods were used for polysaccharides extraction. Then, the obtained crude polysaccharides were separated and purified via column chromatography [28]. After dealing with periodate oxidation, the smith degradation reaction, methylation analysis, partial acid hydrolysis and an enzymatic reaction, the monosaccharide composition and structural characteristics of the polysaccharides were determined via thin-layer chromatography (TLC), high-performance liquid chromatography (HPLC), GC-MS analysis, gel chromatography analysis, nuclear magnetic resonance (NMR) analysis and electrophoresis analysis [26,27,28]. At present, the polysaccharides identified from *Fructus Malvae* mainly include the neutral polysaccharides MVS-I, MVS-IIA, MVS-IIG, the acidic polysaccharides MVS-IIIA, MVS-IVA, MVS-VI and the peptidoglycan MVS-V [25,26,27,28,29,30]. The monosaccharide composition and molar ratio of the neutral polysaccharide MVS-Ⅰ were determined to be l-arabinose:d-galactose:d-glucose = 3:6:7 [26,28], and the monosaccharide composition and molar ratio of the acidic polysaccharide MVS-VI were l-arabinose:d-xylose:d-glucose:l-rhamnose:d-galacturonic acid = 30:15:20:3:2:10 [27]. In addition, the oligosaccharides sucrose and raffinose were also isolated and identified from the dichloromethane extract of *Malva verticillata* seeds [22]. The information and chemical structures are summarized in Table 6 and Figure 7.

### 2.7. Amino Acids

**Figure 8:**
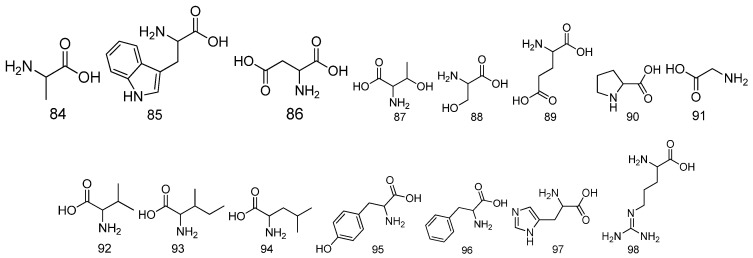
The chemical structures of amino acids identified from *Fructus Malvae*.

For amino acids, 15 amino acids have been detected from *Fructus Malvae* at present (Table 7 and Figure 8). The medicinal powder of *Fructus Malvae* was soaked in water for 12 h, and then, boiled in hot water for half an hour. It was filtered, the filtrates were combined and the supernatant was taken after centrifugation. After that, the supernatant was eluted using a 732 cation-exchange resin. Finally, a total of 14 amino acids were detected using the amino acid automatic analysis and test system [14,18]. Furthermore, tryptophan was the 15th amino acid detected from n-butanol or 90% ethanol extracts of the *Malva verticillata* stem, leaf and seed mixture [31,35]. Additionally, tryptophan showed a synergistic antidiabetic effect together with 3,5,6,9-tetrahydroxy-7-megastigmene in [35].

### 2.8. Other Substances

**Figure 9:**
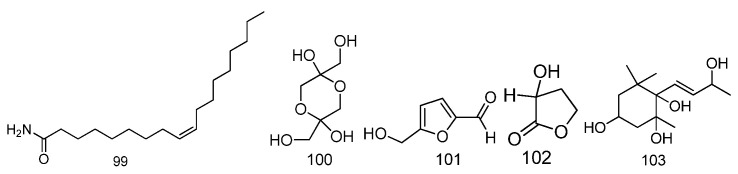
The chemical structures of other substances identified from *Fructus Malvae*.

In addition to the above substances, oleamide, 1,3-dihydroxyacetone dimer, 5-hydroxymethyl furfural, 2-hydroxy-gamma-butyrolactone and other compounds were identified via GC-MS from the water extract of *Fructus Malvae* [23]. The compound 3,5,6,9-tetrahydroxy-7-megastigmene was identified from the n-butanol extract of the *Malva verticillata* stem, leaf and seed mixture [35]. Additionally, 15 trace elements including K, Na, Ca, Mg, Fe, Mn, Zn, Cu, Cr, Se, Pb, Al, Cd, Mo and Ni were also detected from *Fructus Malvae* using an atomic absorption spectrophotometer [36]. The chemical name, molecular formula, medicinal parts and chemical structures are summarized in Table 8 and Figure 9.

## 3. Research Progress on Pharmacological Activity

Shizhen Li recorded the following in the “*Compendium of Materia Medica*”: “Kui, the smell and taste both are light. The character of light and slippery belongs to yang. Therefore, it could promote lactation, reduce swelling and induce abortion. Its roots and leaves have the same function as the seeds”. In the *Chinese Pharmacopoeia* (2020 edition), it is described as follows: “*Fructus Malvae* has the effects of clearing heat, inducing diuresis and reducing swelling”. In addition, *Fructus Malvae* also shows some other pharmacological effects such as having anti-diabetes, antioxidant and anti-tumor properties, the alleviation hair loss, etc. As shown in Table 9.

### 3.1. Diuretic Effect

The diuretic effect is the typical pharmacological effect of *Fructus Malvae*, as reported in *Chinese Pharmacopoeia* (2020 edition) and in the literature. Additionally, it is usually used to treat urinary retention, edema and thirst in a clinical setting [4,31]. Furthermore, heart failure [37,38], liver cirrhosis ascites [39,40], urinary calculi [41,42], hypertensive nephropathy [41] and other diseases often need diuretic drugs for treatment in a clinical setting. With regard to animal experiments, the rat metabolic-cage experiment is often utilized to detect the diuretic effect of drugs [43,44,45]. Additionally, male SD rats [46] or Wistar rats [47] are generally selected. Usually, the rats are fasted for 12–18 h, after pressing the abdomen of the rats to drain the remaining urine in the bladder, 0.9% normal saline or deionized water is administered to form a water-loaded rat model [44,48,49]. Hydrochlorothiazide or furosemide are often selected as positive drugs [47,48], and the urine volume, concentration of electrolytes (sodium ion, chloride ion and potassium ion) and the urine pH are measured to help analyze the diuretic effects [50]. For the rat metabolic-cage diuretic experiment of *Fructus Malvae*, the medicinal powder was refluxed using 70% ethanol, and then, extracted usinf petroleum ether, ethyl acetate, n-butanol and water, respectively, to obtain different solvent extracts [18]. The medication administration groups were given different solvent extracts, and the positive control group was given hydrochlorothiazide [18]. Compared with the negative control group, the results showed that the petroleum ether extract and ethyl acetate extract of *Fructus Malvae* could significantly increase the urine volume of rats. Additionally, the petroleum ether extract also significantly increased the urinary potassium excretion of rats, showing a strong diuretic effect [18].

### 3.2. Anti-Diabetic Effect

At present, the ethyl acetate extract, n-butanol extract, water extract and n-hexane extracts of *Fructus Malvae* show an anti-diabetic effect [32,35]. Additionally, flavonoids including nortangeretin-8-O-β-d-glucuronide, hypolaetin 8-O-β-d-glucuronopyranoside, herbacetin 8-O-β-d-glucuronopyranoside, isoscutellarein 7-O-β-d-glucopyranoside and polysaccharides (including neutral polysaccharide MVS-Ⅰ, peptidoglycan MVS-V and peptidoglycan-enriched fraction MVS-V-C) also showed anti-diabetic activity [29,32]. l-tryptophan and 3,5,6,9-tetrahydroxy-7-megastigmene showed synergistic antidiabetic effect [35]. Most of them were experimentally validated at the whole animal level. Alloxan-induced islet-damage models in zebrafish larvae, type 2 diabetes db/db mice and L6 myotube cells are the common experimental models utilized in anti-diabetes activity studies [24,32,35]. The administration of the method includes soak absorption, oral administration, incubation and intraperitoneal injection [24,29,32,35]. Common detection indicators include fasting blood glucose; body weight; islet size; triglycerides; low-density lipoproteins; high-density lipoproteins; total cholesterol; liver and kidney weight and histopathology; the cytokines TNF-α, IL-1 and IL-6; aspartate aminotransferase; alanine aminotransferase, etc. [51,52].

For the anti-diabetic study of *Fructus Malvae*, zebrafish larvae were placed in a 24-well plate and exposed to 600 μM alloxan solution for 3 h to build the islet cell injury model [32,35]. After that, the zebrafish were treated with n-butanol, ethyl acetate water extractions (10 μg/mL) and the six flavonoids (0.1 μM) separated from *Fructus Malvae* for 12 h. Glimepiride was used as a positive drug in the control group [32]. As result, the nortangeretin-8-O-β-d-glucuronide (25), hypolaetin 8-O-β-d-glucuron opyranoside (27), herbacetin 8-O-β-d-glucuronopyranoside (28) and isoscutellarein 7-O-β-d-glucopyranoside (30) significantly increased the size of the damaged islets. Additionally, compounds **25** and **30** significantly increased the insulin secretion by regulating K_ATP_ channels compared with the model group [32]. 3,5,6,9-tetrahydroxy-7-megastigmene (103) and tryptophan (85) were isolated from the n-butanol extract of *Fructus Malvae* [35]. The zebrafish models were treated with n-butanol extract (10 μg/mL), the two compounds mentioned above and a mixture of them (1 μg/mL) for 12 h [35]. Compared with the model groups after treatment, compound **103** and compound **85** increased the size of the damaged islets by 22.3% and 18.9%, respectively, while the mixture of the two compounds increased the size of the damaged islets by 48.6%, indicating that 3,5,6,9-tetrahydroxy-7-megastigmene (103) and tryptophan (85) obviously exhibit synergistic antidiabetic activity [35]. In another study, the type 2 diabetic mice (db/db) were administrated the n-hexane extract of *Fructus Malvae* (40 mg/kg) for 4 weeks. The result showed that the non-fasting blood glucose and fasting blood glucose of the mice decreased by 17.1% and 23.3%, respectively [24]. Meanwhile, the phosphorylation levels of AMPK and ACC significantly increased. Furthermore, β-sitosterol (31) was the main active compound in this n-hexane extraction [24]. The neutral polysaccharide MVS-I (75) contained in *Fructus Malvae* also showed hypoglycemic activity, according to [29].

Clinically, diabetes is a kind of chronic metabolic disease [53,54], often accompanied by complications such as diabetic nephropathy, diabetic retinopathy, diabetic foot and cardiovascular disease [54,55,56], which are extremely harmful to human health. Exercise, dietary intervention and drug therapy are generally used for the treatment of diabetes [57,58]. In terms of drug treatment, insulin, metformin, glimepiride and other drugs are generally used clinically, but they would also lead to some adverse reactions such as hypotension, obesity, etc. [58]. The use of *Fructus Malvae* in the treatment of diabetes may produce synergistic effects with above western drugs, reducing the corresponding toxic and side-effects. Therefore, further research on the anti-diabetic effect of *Fructus Malvae* would be of great benefit. In addition, the above findings will also contribute to the discovery of new antidiabetic drugs.

### 3.3. Antioxidant Effect

So far, the 90% ethanol extract of *Fructus Malvae* and flavonoids—including nortangeretin-8-O-β-d-glucurono pyranoside, isoscutellarein 8-O-β-d-glucuronopyranoside, hypolaetin 8-O-β-d-glucuronopyranoside, herbacetin 8-O-β-d-glucuronopyranoside, herbacetin 3-O-β-d-glucopyranosyl-8-O-β-d-glucuronopyranoside and isoscutellarein 7-O-d-glucopyranoside—separated and identified from the stem, leaf and seed mixture of *Malva verticillata* L. has shown anti-oxidant activity [31,32]. The DPPH free-radical scavenging test [59,60], ABTS free-radical scavenging test [61,62], total-antioxidant capacity assay (FRAP) [63], oxygen-radical absorbance capacity (ORAC) assay [64] and superoxide scavenging activity assay were used to determine the antioxidant activity of *Fructus Malvae* and its related active ingredients in vitro. DPPH EC50, ABTS EC50, ORAC, SOD EC50, etc. were calculated as the detection indicators.

For the anti-oxidant study of *Fructus Malvae*, in the DPPH free-radical scavenging test, the free-radical scavenging activity of 90% ethanol extract of *Malva verticillata* leaves was 12.62 ± 0.41 mg AAE/g extract, of the stems was 5.15 ± 0.19 mg AAE/g extract and of the seeds was 22.14 ± 0.59 mg AAE/g extract [31]. These results indicated that the seeds of *Malva verticillata* L. (*Fructus Malvae*) had a better DPPH free-radical scavenging activity than the stems and leaves. Meanwhile, in the ABTS free-radical scavenging test and total-antioxidant capacity assay (FRAP)—wherein antioxidant activity was evaluated by measuring the absorbance value of the ABTS free-radical working solution at 734 nm and the absorbance value of the FRAP working solution at 539 nm, respectively—the 90% ethanol extract of the leaves showed better antioxidant activity than the stems and seeds [31]. In another study, nortangeretin-8-O-β-d-glucuronopyranoside, isoscutellarein 8-O-β-d-glucuronopyranoside, hypolaetin 8-O-β-d-glucuronopyranoside, herbacetin 8-O-β-d-glucuronopyranoside, and herbacetin 3-O-β-d-glucopyranosyl-8-O-β-d-glucuronopyranoside showed significant antioxidant activity in the ABTS, ORAC, SOD tests [32]. Additionally, the results indicated that the 8-O-glucuronide attached to a flavonoid moiety was a key structure of the antioxidant activity. Due to the presence of a 1,2,3-trihydroxy benzene moiety in the flavonoid A-ring and a 1,2-dihydroxy benzene moiety in the flavonoid B-ring, nortangeretin-8-O-β-d-glucuronopyranoside and hypolaetin 8-O-β-d-glucuronopyranoside showed especially high antioxidant activity in the ABTS and ORAC assays [32].

In a clinical setting, it is widely accepted that antioxidant effects are mostly related to age-related diseases [65], such as cardiovascular disease [66,67], non-alcoholic fatty liver disease [67], vascular dementia [68], Graves’ ophthalmopathy [69], cancer [70,71], diabetes [72], etc. ROS, including hydrogen peroxide (H_2_O_2_), hydroxyl radical (•OH), singlet oxygen (1O_2_), superoxide (O_2_^2−^), etc., is a group of unstable molecules produced by various cells in the human body. These free radicals could take part in human metabolism, immunity, growth, differentiation and many other homeostatic processes [73,74,75]. The cells and tissues will be damaged oxidatively and stay in a pathological state when ROS is excessively produced in the body [76]. At that time, antioxidative drugs are needed to resist the peroxidative effect of ROS on the human body. The above research and discoveries would be very beneficial and promising for the discovery of new antioxidative drugs.

### 3.4. Antitumor Effect

The ethyl acetate extract, n-butanol extract, water extract and 17 glycerides identified from *Fructus Malvae* or the stem, leaf and seed mixture of *Malva verticillata* L. show significant anti-tumor activity [33,34]. They have all been verified at the cellular level. Splenocytes, natural killer (NK) cells, human liver cancer cells (HepG2) [77], human gastric cancer cells (AGS), human colorectal cancer cells (HCT) and human non-small-cell lung cancer calls (A549) [77,78] were selected as the experimental models to verify the anti-tumor activity of *Fructus Malvae.* Splenocyte proliferation ability, natural killer (NK) cell activity, AGS cell apoptosis percentage, and the expression of the apoptosis proteins PARP, Cleaved APRP, Caspase-3, Cleaved Caspase-3, Bcl-2, Bax, β-actin, etc. were detected as indicators in the study [33,34]. In addition to the above cell experiments, tumor-bearing mice have also been used to verify anti-tumor activity at the animal experimental level in other studies [79,80,81]. Additionally, tumor volume and mass; thymus index; spleen index; the serum cytokines IL-2, IL-4 and TNF-α; IFN-ɤ levels and tumor histopathology could be detected as detection indicators [82]. Additionally, usually, splenocyte and tumor cells would be cultured in RPMI1640 medium or in DMEM medium, which contains 10% fetal bovine serum (FBS) and 1% penicillin-streptomycin [83,84]. The proliferation ability or the viability of tumor cells can be measured using tetramethylazolyl blue (MTT method) or Cell Counting Kit 8 (CCK8) [83]. The apoptosis of tumor cells can be measured using a Tali apoptosis assay kit. The migration ability of tumor cells can be measured using the scratch method or transwell-migration method. Western blot is generally used to measure the expression of apoptosis, its pathway and other related proteins in tumor cells [85].

For the anti-tumor study of *Fructus Malvae*, the monoacylglycerides (2S)-1-O-palmitoyl glyceride (compound **34**), (2S)-1-O-stearoyl glyceride (compound **35**) and (2S)-1-O-linolenoyl glyceride (compound **36**), separated and identified from the stem, leaf and seed mixture of *Malva verticillata*, significantly enhanced the proliferation ability of splenocytes and the activity of natural killer cells against tumor cells at 10 μM [33]. Meanwhile, diacylglyceride (2S)-1,2-di-O-linoleoyl glyceride showed weaker activity. The results indicated that monoacylglycerides exhibited stronger antitumor activity than diacylglycerides. Additionally, the longer the carbon chain of fatty acids, the better the antitumor activity in monoacylglycerides [33]. Additionally, unsaturated fatty acids showed better activity than saturated fatty acids [33]. In another study, 13 glycosylglycerides identified from the stem, leaf and seed mixture of *Malva verticillata* L. showed cytotoxicity against HepG2, AGS, HCT-15 and A549 human cancer cells in in vitro cell experiments [34]. Among them, the chemical structures of two glycosylglycerides (compounds **38** and **39**) contained a unique glycosyl group (6-deoxy-6-sulfo)-α-d-glucopyranosyl) and they showed especially significant cytotoxicity to AGS tumor cell, increasing the apoptosis of AGS cells and affecting the expression of apoptotic proteins [34].

In a clinical setting, surgery, chemotherapy, interventional therapy, immunization and targeted therapy are often used for the treatment of cancers/tumors [86,87]. Cytotoxic drugs such as cyclophosphamide, hormonal drugs such as exemestane, and monoclonal antibodies such as rituximab are often used in the treatment process [88]. However, these therapeutic drugs are often accompanied by serious toxic and side effects, easily resulting in liver and kidney damage [89,90], hair loss [91], drug resistance [87], etc. A study developed by He Zhu et al. found that the combination of traditional Chinese medicine and western medicine can produce a synergistic anti-tumor effect and reduce the toxic and side-effects of the drug [92]. *Fructus Malvae* may be beneficial in such an application. Furthermore, the above findings would also be beneficial for the discovery of new anti-tumor drugs.

### 3.5. Treatment of Hair Loss

Extracts of 95% ethanol, ethanol, n-hexane and dichloromethane of *Fructus Malvae* showed pharmacological activity in treating hair loss [21,22]. Linoleic acid and myristoleic acid were two active compounds. They were all verified at the cellular level by human dermal papilla cells (DPCs) [21,22]. A cell proliferation ability, the expression of Wnt/β-catenin signaling pathway proteins and cell growth factor were detected as detection indicators. Additionally, mice can also be used. For this, the hair on the back of the mice is removed to build a model. The growth rate and appearance of the hair, the number of hair follicles, the hormone levels in the mouse blood, the growth factors in the mouse skin cells, and the histological morphology can be observed or measured to verify the pharmacological activity in the treatment of hair loss.

For the hair-loss-treatment study of *Fructus Malvae*, a study confirmed that linolenic acid can activate the Wnt/β-catenin signaling pathway and increase the expression of cyclins such as cyclinD1, CDK2 and the cell growth factors VEGF, IGF-1, etc. in a dose-dependent manner [21]. When the administration concentration increased from 10 μg/mL to 30 μg/mL, the HFDPC cell proliferation rate increased by 21.46% [21], while oleic acid showed no relevant pharmacological activity. In addition, another research team found that the dichloromethane extract of *Fructus Malvae* and the active compound myristoleic acid separated from it also showed a therapeutic effect on hair loss [22]. Similar to the effect of linolenic acid, myristoleic acid can activate the Wnt/β-catenin signaling pathway and promote the proliferation of DPCs cell in the treatment of hair loss [22].

In a clinical setting, due to the advancement of technology, the accelerated pace of life, life pressure, work pressure and unhealthy work and diet habits, hair loss has become an important health problem faced by people. According to a population epidemiological survey, about 70% of the population in China is suffering from hair loss, and the phenomenon of hair loss shows a serious trend in youth [93]. Clinically, alopecia refers to a skin disorder characterized by hair loss, and includes androgenetic alopecia (seborrheic alopecia), alopecia areata and congenital alopecia [93,94]. Minoxidil and finasteride are often used to treat androgenetic alopecia, while steroids and retinoic acid are often used to treat alopecia areata [94]. However, the external use of minoxidil can easily cause dermatitis and increases the amount of body hair; finasteride can easily cause hormonal disorders and sexual dysfunction in the human body; and steroids can easily lead to scalp shrinkage and full-moon face [93,94]. The findings above indicate that *Fructus Malvae* may become a new choice for the treatment of hair loss.

### 3.6. Other Pharmacological Effects

In addition to the pharmacological effects above, *Fructus Malvae* also shows the potential ability to treat pathological bone disease [23], enhancing reticuloendothelial system activity and increasing anti-complement activity [25,26,27,28,29,30]. A study identified 14 compounds using GC-MS analysis from the water extract of *Fructus Malvae*. The results indicated that the water extract could inhibit the RANKL signaling pathway, and further inhibited osteoclastogenesis and bone resorption. Therefore, *Fructus Malvae* could also be used as a supplementary alternative drug for the treatment of pathological bone diseases [23]. Most of the polysaccharide components reported in *Fructus Malvae* showed the effect of enhancing reticuloendothelial system activity and anti-complement activity. More detailed information can be found in references [20,25,26,27,28,29].

## 4. Summary and Discussion

The outbreak of the COVID-19 pandemic has brought great harm and challenges to the economic development and public health services of many countries and regions [95,96]. In China, traditional Chinese medicine is used in the treatment of COVID-19 and has obtained remarkable success [97]. The Lianhua Qingwen capsule is one of the most widely reported [98]. Additionally, the discovery of artemisinin in Artemisia annua has reduced the incidence of malaria around the world [3]. Therefore, it is becoming urgent and important to establish a novel method of conducting systematic research on Chinese herb medicine, to bring great benefits to human health all over the world.

*Fructus Malvae*, a kind of Chinese herb medicine, refers to the dried and ripe fruit of *Malva verticillata* L. [4]. So far, certain studies have been performed at home and abroad that are associated with the chemical composition and biological activity of *Fructus Malvae*. The chemical composition of *Fructus Malvae* is varied, mainly including 9 acid compounds (phenolic acids and fatty acids), 21 flavonoids, 3 sterols, 17 glycerides, 24 kinds of volatile oil, 9 kinds of polysaccharide, 15 kinds of amino acid and 5 other compounds. The above compounds and different solvent extracts of *Fructus Malvae* have shown various pharmacological activities according to the reports, including having diuretic, anti-diabetic, anti-oxidative and anti-tumor effects, treating hair loss, etc.

The mixture of the stems, leaves and seeds of *Malva verticillata* L. was extracted using 80% methanol at room temperature for 24 h. The extract was filtered and concentrated under reduced pressure to obtain methanol extract, which was dispersed in water, and then, extracted using ethyl acetate and n-butanol in sequence [32,33,34]. Six flavonoids, including nortangeretin-8-O-β-d-glucuronopyranoside (compound **25**), isoscutellarein 8-O-β-d-glucuronopyranoside (compound **26**), hypolaetin 8-O-β-d-glucurono pyranoside (compound **27**), herbacetin-8-O-β-d-glucuronopyranoside (compound **29**) and isoscutellarein 7-O-β-d-glucopyranoside (compound **30**) were separated and identified from the water extract [32]. Compound 25 is a new compound, and the 5,6,7,8-tetrahydroxy group and the 8-O-glucuronide attached to the A ring of the flavonoid moiety are rarely reported in plants [32]. A total of 17 glycerides were separated from n-butanol extracts [33,34] (compound **34**–**50**), and among them, there are 13 glycosylglycerides (compounds **38**–**50**) [34]. Additionally, tryptophan (compound **85**) and 3,5,6,9-tetrahydroxy-7-megastigmene (103) were also identified from n-butanol extracts [35]. Among them, compounds **34**, **35** and **36** are monoacylglycerides, while compound **37** is a diacylglyceride [33]. Studies have confirmed that monoacylglycerides are more active than diacylglycerides. Additionally, among monoacylglycerides, the longer the fatty-acid carbon chain, the better the activity, and the unsaturated fatty acid is more active than the saturated fatty acid [33]. 1-O-galactopyranosyl-3-O-isostearoyl glyceride (compound **42**) is a novel glycosylglyceride compound. 1-O-(6-deoxy-6-sulfo)-glucopyranosyl-2-O-linolenoyl-3-O-palmitoyl glyceride and 1-O-(6-deoxy-6-sulfo)-glucopyranosyl-2,3-di-O-linolenoyl glyceride (compounds **38**, **39**) are two sulfoquinovosyl glycerides, and both of them contain a unique chemical structure “(6-deoxy-6-sulfo)-a-d-glucopyranose” which is rarely reported in plants [34]. Tryptophan (**85**), the precursor of 5-hydroxytryptophan (5-HT) and melatonin, is a kind of essential amino acid [35]. The ratio of compounds **85** and **103** in the n-butanol extract was 1.96:1. *Fructus Malvae* was extracted using ethanol, and then, extracted using dichloromethane, ethyl acetate, water, n-hexane and other solvents. Myristoleic acid (compound **8**) was isolated and identified from dichloromethane extract [22] and linolenic acid was isolated and identified from n-butanol extract [21], which are two polyunsaturated fatty-acid compounds.

In terms of pharmacological activity, the diuretic activity of *Fructus Malvae* was studied using the rat metabolic-cage diuretic test [18]. The petroleum ether and ethyl acetate extract of *Fructus Malva* significantly increased the urine output of water-loaded rats. Additionally, the petroleum ether extract also significantly increased their urinary potassium content [18]. In antidiabetic studies, the ethyl acetate, n-butanol, water and n-hexane extracts of *Fructus Malva* (or of the mixture of stems, leaves and seeds) showed antidiabetic activity [32,35]. The flavonoids nortangeretin-8-O-β-d-glucuronide, hypolaetin8-O-β-d-glucuronopyranoside, herbacetin 8-O-β-d-glucuronopyranoside and isoscutellarein 7-O-β-d-glucopyranoside were able to significantly increase the size of alloxan-injured zebrafish islets, and the study also confirmed that nortangeretin-8-O-β-d-glucuronide and isoscutellarein 7-O-β-d-glucopyranoside were able to block K+ ions channel in islet β cells to increase the size of alloxan-injured zebrafish islets [32]. In addition, 3,5,6,9-tetrahydroxy-7-megastigmene and tryptophan in *Fructus Malva* also showed significant synergistic antidiabetic activity [35]. When given 1 μg/mL of two compounds, the size of damaged islets in zebrafish was increased by 22.3% and 18.9%, respectively, while their mixture increased the size of damaged islets by 48.6% [35]. The sterol compound β-sitosterol, the neutral polysaccharide MVS-I, the peptidoglycan MVS-V and the peptidoglycan-enriched fraction MVS-V-CH of the polysaccharides also showed activity in the treatment of diabetes [24,29]. For the experimental animal model, in addition to the alloxan-induced zebrafish islet cell-damage model [32,35], a db/db mouse model of type 2 diabetes was also used [24]. In antioxidant research, the DPPH free-radical scavenging assay, the ABTS free-radical scavenging assay, the FRAP total-antioxidant capacity-measurement experiment, the ORAC oxygen-radical absorption capacity-measurement experiment and the SOD superoxide dismutase scavenging assay were used to determine the antioxidant capacity of the of chemical substances in mallow fruit in vitro [31,32]. The 70% ethanol-extracted fraction and six flavonoids identified from *Fructus Malvae* showed strong antioxidant activity in vitro [31,32]. In a study of anti-tumor activity, the anti-tumor activity of *Fructus Malva* was verified using immune cells such as splenocytes, natural killer cells (NK), and human tumor cells such as HepG2, AGS, HCT-15 and A549 [33,34]. The glyceride compounds (2S)-1-O-palmitoyl glyceride, (2S)-1-O-stearoyl glyceride and (2S)-1-O-linolenoyl glyceride can significantly increase the proliferation ability of splenocytes and the activity of natural killer cells against tumor cells [33]. Thirteen glycosylglycerides have shown antitumor activity against HepG2, AGS, HCT-15 and A549 human tumor cells [34]. 1-O-(6-deoxy-6-sulfo)-glucopyranosyl-2-O-linolenoyl-3-O-palmitoyl glyceride, 1-O-(6-deoxy-6-sulfo)-glucopyranosyl-2,3-di-O-linolenoyl glyceride and 1-O-6′-O-(-galactopyranosyl)-galactopyranosyl-2,3-di-O-palmitoyl glyceride have particularly significant effects on AGS tumor cells, accelerating tumor cell apoptosis and influencing the expression of apoptosis proteins such as PARR, caspase-3, Bcl-2, Bax and β-actin [34]. In studies of the treatment of hair loss, the 95% ethanol/ethanol, n-hexane and dichloromethane extracts showed a therapeutic effect on hair loss [21,22]. Linolenic acid and myristoleic acid, two kind of fatty acid in *Fructus Malva*, significantly activated the Wnt/β-catenin signaling pathway and promoted the proliferation of human dermal papilla cell DPCs, which could become a new choice for the treatment of hair loss [21,22].

**Figure 10:**
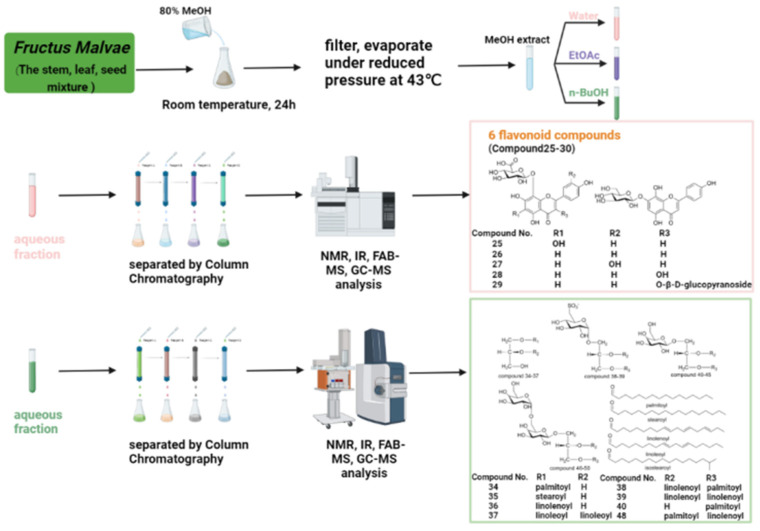
The typical chemical composition separated and identified from *Fructus Malvae*.

**Figure 11:**
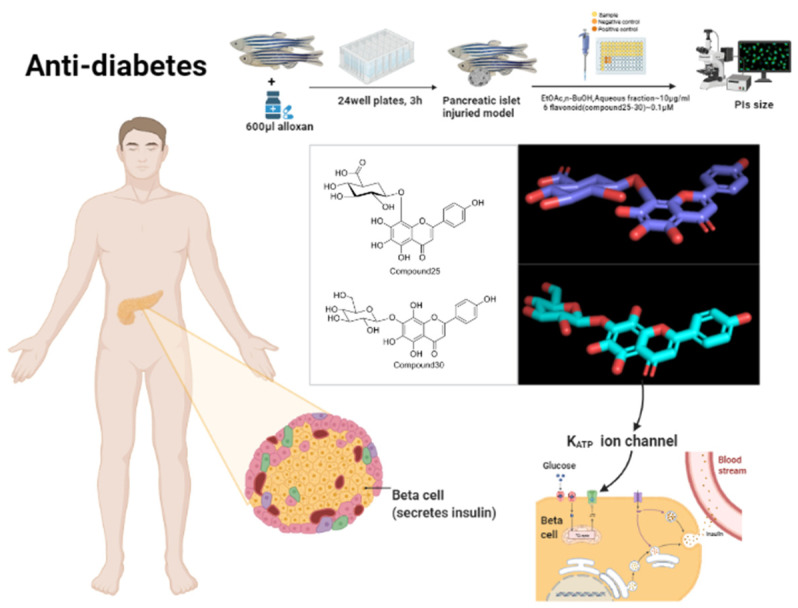
The anti-diabetic effect of 2 flavonoids identified from *Fructus Malvae*.

With the improvement of industrialization, lifestyle changes, unhealthy eating habits, obesity/overweight and other factors, the incidence of diabetes is increasing year by year [99]. Globally, there are 382 million people, about 8.3% of the population, suffering from diabetes. Furthermore, diabetes has become the main cause of death for people under 60 years old [99]. Therefore, it is of great clinical value and prospect to further study the anti-diabetes effect of *Fructus Malvae*. So far, there have been six flavonoids (compounds **25–30**) isolated and identified from the water extract of *Fructus Malvae*, showing potential antidiabetic effects (Figure 10) [32]. Among them, nortangeretin-8-O-β-d-glucuronopyranoside (compound **25**) is a new compound. The 5,6,7,8-tetrahydroxyl and 8-O-glucuronide attached to the A ring of the flavonoid group are novel structures that are rarely reported in plants [32]. Experiments have confirmed that nortangeretin-8-O-β-d-glucuronopyranoside can significantly recover alloxan-induced islet damage and block the K+ channel of islet β-cells in zebrafish. What’s more, so dose isoscutellarein 7-O-β-d-glucopyranoside (compound **30**) [32] (as shown in Figure 11). They could be selected as important candidate compounds for a diabetes-treatment drug. Regarding methodological design, male SD rats can also be used instead of the zebrafish mentioned above. They should be fed high-fat and high-sugar diets for 4–6 weeks, and 1% streptozotocin or alloxan should be injected via intraperitoneal injection to build a type 2 diabetes rat model [100,101,102]. Furthermore, db/db mice could also be used directly to verify the anti-diabetic effect of *Fructus Malvae* [103,104].

**Figure 12:**
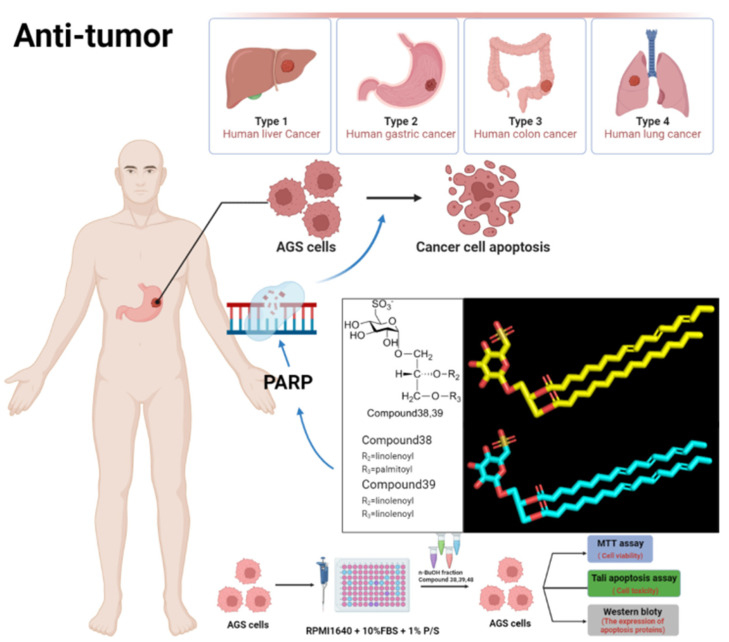
The anti-tumor effect of 2 glycosylglycerides identified from *Fructus Malvae*.

Cancer is the leading cause of death in people before the age of 70. According to the “2020 Global Cancer Statistics” released by the American Cancer Society, there were 19.3 million new cancer cases and 10 million cancer deaths worldwide in 2020. There were 4.136 million new cancer cases in China, accounting for 21.0% of the global new cancer cases. The top five cancers, ranked by mortality rate, are lung cancer, colorectal cancer, liver cancer, gastric cancer and female breast cancer [105]. Therefore, it is necessary to further research the anticancer effect of *Fructus Malva*. Among the 17 glyceride compounds (compounds **34**–**50**) [33,34] identified from the n-butanol extract of *Fructus Malva*, compounds **38**–**50** are glycosylglyceride compounds (Figure 10), which show significant cytotoxicity against the human hepatoma cell HepG2, the human gastric cancer cell AGS, the human colorectal cancer cell HCT-15 and the human non-small-cell lung cancer cell A549 [34]. The glycosylglycerides (2S)-1-O-(6-deoxy-6-sulfo)-α-d-glucopyranosyl-2-O-linolenoyl-3-O-palmitoyl glyceride and (2S)-1-O-(6-deoxy-6-sulfo)-α-d-glucopyranosyl-2,3-di-O-linolenoyl glyceride (compounds **38**, **39**) were isolated from *Fructus Malva* for the first time and have rarely been reported in plants before. Both compounds contained a unique chemical structure (6-deoxy-6-sulfo)-α-d-glucopyranosyl, which also rarely occurred before. They showed significant cytotoxicity to AGS tumor cells. They accelerated the apoptosis of AGS cells, and significantly affected the expression of apoptotic proteins such as PARP, caspase-3, Bcl-2, Bax and β-actin [34] (Figure 12). They could be further studied as candidate compounds for antitumor drugs. At the same time, monoacylglycerides compounds **34**, **35** and **36** significantly enhanced the proliferation ability of spleen cells and the antitumor activity of natural killer cells, and their activity was stronger than that of the diacylglyceride 1,2-di-O-linoleoyl glyceride (compound **37**) [33]. Additionally, for the methodological design, in addition to verification at the cellular level, tumor-bearing mice could also be used in research [106]. By evaluating the tumor volume and mass, thymus index, spleen index, serum cytokine levels, tumor histopathology, etc. [82], the anti-tumor activity of *Fructus Malva* and its active ingredients could be further verified at the level of animal experiments.

At present, some drug standards, including the *Chinese Pharmacopoeia* (2020 edition), the *Traditional Chinese Medicine Processing Specifications of Jiangxi Province* (2008 edition), the *Traditional Chinese Medicine Processing Specifications of Hunan Province* (2010 edition), the *Traditional Chinese Medicine Processing Specifications of Guangxi Province* (2007 edition), *Traditional Chinese Medicine Processing Specifications of Gansu Province* (2009 edition), etc. have standardized the production and inspection processes of *Fructus Malvae*. However, so far, there have still been serious mixed-sales and mixed-use phenomena for *Fructus Malva* and its confounded medicinal material, *Abutili Semen*, in the medicinal material market and clinical use, bringing certain obstacles to further research on the medicinal value of *Fructus Malvae*. At the same time, although *Fructus Malva* is widely used in clinical settings, its application mainly focuses on the classic pharmacological activity and diuretic effect of *Fructus Malvae*. Other promising pharmacological activities such as anti-diabetic effect, anti-tumor effect and treatment of hair loss still stay at the level of basic research, which are the focus of current research. This is not only a challenge, but also an opportunity for the development of the medicinal value of *Fructus Malvae*.

## Figures and Tables

**Table 1:** The acid compounds identified from *Fructus Malvae*.

No.	Compound Name	Molecular Formula	Medicinal Parts	References
**1**	Caffeic acid	C_9_H_8_O_4_	The fruit of *Malva verticillata* L.	[7,11,12,13,14,15]
**2**	Ferulic acid	C_10_H_10_O_4_	The fruit of *Malva verticillata* L.	[7,15,16,17]
**3**	Quinic acid	C_7_H_12_O_6_	The stem, leaf and seed mixture of *Malva verticillata* L.	[31]
**4**	9,12,13-trihydroxy-octadecadienoic acid	C_18_H_32_O_5_	The stem, leaf and seed mixture of *Malva verticillata* L.	[31]
**5**	9,12,13-trihydroxy-octadecenoic acid	C_18_H_34_O_5_	The stem, leaf and seed mixture of *Malva verticillata* L.	[31]
**6**	Linolenic acid	C_18_H_30_O_2_	The stem, leaf and seed mixture of *Malva verticillata* L.	[21,31]
**7**	Oleic acid	C_18_H_34_O_2_	The seed of *Malva verticillata* L.	[21]
**8**	Myristoleic acid	C_14_H_26_O_2_	The seed of *Malva verticillata* L.	[22]
**9**	Palmitic acid	C_16_H_32_O_2_	The seed of *Malva verticillata* L.	[23]

*Fructus Malvae* refers to the fruit or seed of *Malva verticillata* L.

**Table 2:** The flavonoids identified from *Fructus Malvae*.

No.	Chemical Name	Molecular Formula	Medicinal Parts	Reference
**10**	Myricetin-3-hexoside-glucuronide	C_27_H_28_O_19_	The stem, leaf and seed mixture of *Malva verticillata* L.	[31]
**11**	Quercetin-3-O-hexoside-glucuronide	C_27_H_28_O_18_	The stem, leaf and seed mixture of *Malva verticillata* L.	[31]
**12**	Epigallocatechin	C_15_H_14_O_7_	The stem, leaf and seed mixture of *Malva verticillata* L.	[31]
**13**	Hyperin	C_21_H_20_O_12_	The stem, leaf and seed mixture of *Malva verticillata* L.	[31]
**14**	Kaempferol-3-O-rutinoside	C_27_H_30_O_15_	The stem, leaf and seed mixture of *Malva verticillata* L.	[31]
**15**	Kaempferol-3-O-glucoside	C_21_H_20_O_11_	The stem, leaf and seed mixture of *Malva verticillata* L.	[31]
**16**	Quercitrin	C_21_H_20_O_11_	The stem, leaf and seed mixture of *Malva verticillata* L.	[31]
**17**	Quercetin-3-O-glucuronide	C_21_H_18_O_13_	The stem, leaf and seed mixture of *Malva verticillata* L.	[31]
**18**	Catechin	C_15_H_14_O_6_	The stem, leaf and seed mixture of *Malva verticillata* L.	[31]
**19**	Kaempferol (or luteolin)-3-O-glucuronid	C_21_H_18_O_12_	The stem, leaf and seed mixture of *Malva verticillata* L.	[31]
**20**	Kaempferide-3-glucuronide	C_22_H_20_O_12_	The stem, leaf and seed mixture of *Malva verticillata* L.	[31]
**21**	Biochanin A	C_16_H_12_O_5_	The stem, leaf and seed mixture of *Malva verticillata* L.	[31]
**22**	Rubone	C_20_H_22_O_7_	The stem, leaf and seed mixture of *Malva verticillata* L.	[31]
**23**	Robinetin trimethyl ether	C_18_H_16_O_7_	The stem, leaf and seed mixture of *Malva verticillata* L.	[31]
**24**	Rutin	C_27_H_30_O_16_	The fruit of *Malva verticillata* L.	[18]
**25**	Nortangeretin-8-O-β-d-glucuronide	C_21_H_18_O_13_	The stem, leaf and seed mixture of *Malva verticillata* L.	[32]
**26**	Isoscutellarein 8-O-glucuronopyranoside	C_21_H_18_O_12_	The stem, leaf and seed mixture of *Malva verticillata* L.	[32]
**27**	Hypolaetin 8-O-glucuronopyranoside	C_21_H_18_O_13_	The stem, leaf and seed mixture of *Malva verticillata* L.	[32]
**28**	Herbacetin 8-O-glucuronopyranoside	C_21_H_18_O_13_	The stem, leaf and seed mixture of *Malva verticillata* L.	[32]
**29**	Herbacetin 3-O-glucopyranosyl-8-O-glucuronopyranoside	C_27_H_28_O_18_	The stem, leaf and seed mixture of *Malva verticillata* L.	[32]
**30**	Isoscutellarein 7-O-glucopyranoside	C_21_H_20_O_11_	The stem, leaf and seed mixture of *Malva verticillata* L.	[32]

*Fructus Malvae* refers to the fruit or seed of *Malva verticillata* L.

**Table 3:** The sterol compounds identified from *Fructus Malvae*.

No.	Chemical Name	Molecular Formula	Medicinal Parts	References
**31**	β-sitosterol	C_29_H_50_O	The seed of *Malva verticillata* L.	[18,22,23,24]
**32**	Verticilloside	C_50_H_80_O_22_	The seed of *Malva verticillata* L.	[22]
**33**	Daucosterol	C_35_H_60_O_6_	The seed of *Malva verticillata* L.	[18,22]

*Fructus Malvae* refers to the fruit or seed of *Malva verticillata* L.

**Table 4:** The Glycerides identified from *Fructus Malvae*.

No.	Chemical Name	Molecular Formula	Medicinal Parts	Reference
**34**	1-O-palmitoyl glyceride	C_19_H_38_O_4_	The stem, leaf and seed mixture of *Malva verticillata* L.	[33]
**35**	1-O-stearoyl glyceride	C_21_H_42_O_4_	The stem, leaf and seed mixture of *Malva verticillata* L.	[33]
**36**	1-O-linolenoyl glyceride	C_21_H_36_O_4_	The stem, leaf and seed mixture of *Malva verticillata* L.	[33]
**37**	1,2-di-O-linoleoyl glyceride	C_39_H_68_O_5_	The stem, leaf and seed mixture of *Malva verticillata* L.	[33]
**38**	1-O-(6-deoxy-6-sulfo)-glucopyranosyl-2-O-linolenoyl-3-O-palmitoyl glyceride	C_43_H_75_O_12_S^−^	The stem, leaf and seed mixture of *Malva verticillata* L.	[34]
**39**	1-O-(6-deoxy-6-sulfo)-glucopyranosyl-2,3-di-O-linolenoyl glyceride	C_45_H_73_O_12_S^−^	The stem, leaf and seed mixture of *Malva verticillata* L.	[34]
**40**	1-O-galactopyranosyl-3-O-palmitoyl glyceride	C_25_H_48_O_9_	The stem, leaf and seed mixture of *Malva verticillata* L.	[34]
**41**	1-O-galactopyranosyl-3-O-stearoyl glyceride	C_27_H_52_O_9_	The stem, leaf and seed mixture of *Malva verticillata* L.	[34]
**42**	1-O-galactopyranosyl-3-O-isostearoyl glyceride	C_27_H_52_O_9_	The stem, leaf and seed mixture of *Malva verticillata* L.	[34]
**43**	1-O-galactopyranosyl-3-O-linolenoyl glyceride	C_27_H_46_O_9_	The stem, leaf and seed mixture of *Malva verticillata* L.	[34]
**44**	1-O-galactopyranosyl-2,3-di-O-palmitoyl glyceride	C_41_H_78_O_10_	The stem, leaf and seed mixture of *Malva verticillata* L.	[34]
**45**	1-O-galactopyranosyl-2,3-di-O-linolenoyl glyceride	C_45_H_74_O_10_	The stem, leaf and seed mixture of *Malva verticillata* L.	[34]
**46**	1-O-6′-O-(-galactopyranosyl)-galactopyranosyl-3-O-palmitoyl glyceride	C_31_H_58_O_14_	The stem, leaf and seed mixture of *Malva verticillata* L.	[34]
**47**	O-6′-O-(-galactopyranosyl)-galactopyranosyl-3-O-2-linolenoyl glyceride	C_33_H_56_O_14_	The stem, leaf and seed mixture of *Malva verticillata* L.	[34]
**48**	1-O-6′-O-(-galactopyranosyl)-galactopyranosyl-2,3-di-O-palmitoyl glyceride	C_47_H_88_O_15_	The stem, leaf and seed mixture of *Malva verticillata* L.	[34]
**49**	1-O-(6-O-galactopyranosyl)-galactopyranosyl-2-O-stearolyl-3-O-linolenoyl glyceride	C_51_H_90_O_15_	The stem, leaf and seed mixture of *Malva verticillata* L.	[34]
**50**	1-O-(6-O-galactopyranosyl)-galactopyranosyl-2,3-di-O-linolenoyl glyceride	C_51_H_84_O_15_	The stem, leaf and seed mixture of *Malva verticillata* L.	[34]

*Fructus Malvae* refers to the fruit or seed of *Malva verticillata* L.

**Table 5:** The volatile oils identified from *Fructus Malvae*.

No.	Chemical Name	Molecular Formula	Medicinal Parts	References
**51**	1,1-dichloro-2-hexyl-Cyclopropan	C_9_H_16_Cl_2_	The fruit of *Malva verticillata* L.	[19,20]
**52**	3-(Prop-2-enoyloxy)dodecane	C_15_H_28_O_2_	The fruit of *Malva verticillata* L.	[19,20]
**53**	1-(ethenyloxy)-pentane	C_7_H_14_O	The fruit of *Malva verticillata* L.	[19,20]
**54**	1-methyl-6,7-Dioxabicyclo[3.2.1]octane	C_7_H_12_O_2_	The fruit of *Malva verticillata* L.	[19,20]
**55**	2-methyl-5-(1-methylethenyl)-Cyclohexanol	C_10_H_18_O	The fruit of *Malva verticillata* L.	[19,20]
**56**	trans-1,2-Cyclopentanediol	C_5_H_10_O_2_	The fruit of *Malva verticillata* L.	[19,20]
**57**	3, 5-Octadien-2-ol	C_10_H_18_O	The fruit of *Malva verticillata* L.	[19,20]
**58**	(Z)-2-Octen-2-ol	C_8_H_16_O	The fruit of *Malva verticillata* L.	[19,20]
**59**	Nona-2-en-1-ol	C_9_H_18_O	The fruit of *Malva verticillata* L.	[19,20]
**60**	(E)-2,6-Dimethyl-3,5,7-octatriene-2-ol	C_10_H_16_O	The fruit of *Malva verticillata* L.	[19,20]
**61**	(1S)-1,7,7-trimethyl-Bicyclo[2.2.1] heptan-2-one	C_10_H_16_O	The fruit of *Malva verticillata* L.	[19,20]
**62**	Z,Z-2,5-Pentadecadien-1-ol	C_15_H_28_O	The fruit of *Malva verticillata* L.	[19,20]
**63**	Dihydro-4,4-dimethyl-2(3H)-Furano	C_6_H_10_O_2_	The fruit of *Malva verticillata* L.	[19,20]
**64**	Z-1,9-Hexadecadiene	C_16_H_30_	The fruit of *Malva verticillata* L.	[19,20]
**65**	(E, E)-2,4-Decadiene	C_10_H_18_	The fruit of *Malva verticillata* L.	[19,20]
**66**	Hexanoic acid	C_16_H_12_O_2_	The fruit of *Malva verticillata* L.	[19,20]
**67**	2-(Prop-2-enoytoxy) tetradecane	C_17_H_32_O_2_	The fruit of *Malva verticillata* L.	[19,20]
**68**	5-hexyldihydro-2(3H)-Furanone	C_10_H_18_O_2_	The fruit of *Malva verticillata* L.	[19,20]
**69**	7-Methyl-Z-tetradecen-1-ol acetate	C_17_H_32_O_2_	The fruit of *Malva verticillata* L.	[19,20]
**70**	Dibutylphthalate	C_16_H_22_O_4_	The fruit of *Malva verticillata* L.	[19,20]
**71**	2,5-dihydro-1-nitroso-1H-Pyrrole	C_4_H_6_N_2_O	The fruit of *Malva verticillata* L.	[19,20]
**72**	5-(Prop-2-enoyloxy)pentadecane	C_18_H_34_O_2_	The fruit of *Malva verticillata* L.	[19,20]
**73**	(E)-2-Octenal	C_8_H_14_O	The fruit of *Malva verticillata* L.	[19,20]
**74**	(Z)-2-Nonenal	C_9_H_16_O	The fruit of *Malva verticillata* L.	[19,20]

*Fructus Malvae* refers to the fruit or seed of *Malva verticillata* L.

**Table 6:** The polysaccharides identified from *Fructus Malvae*.

No.	Chemical Name	Molecular Formula	Medicinal Parts	References
**75**	MVS-I	--	The seed of *Malva verticillata* L.	[25,26,28,29]
**76**	MVS-IIA	--	The seed of *Malva verticillata* L.	[25,26,29]
**77**	MVS-IIG	--	The seed of *Malva verticillata* L.	[25,26,29]
**78**	MVS-IIIA	--	The seed of *Malva verticillata* L.	[25,26,29]
**79**	MVS-IVA	--	The seed of *Malva verticillata* L.	[25,26,29,30]
**80**	MVS-VI	--	The seed of *Malva verticillata* L.	[25,26,27,29]
**81**	MVS-V	--	The seed of *Malva verticillata* L.	[25,26,29]
**82**	Sucrose	C_12_H_22_O_11_	The seed of *Malva verticillata* L.	[22]
**83**	Raffinose	C_18_H_32_O_16_	The seed of *Malva verticillata* L.	[22]

*Fructus Malvae* refers to the fruit or seed of *Malva verticillata* L.

**Table 7:** The amino acids identified from *Fructus Malvae*.

No.	Chemical Name	Molecular Formula	Medicinal Parts	References
**84**	d-alanine	C_3_H_7_NO_2_	The seed of *Malva verticillata* L.	[14,18,23]
**85**	tryptophan	C_11_H_12_N_2_O_2_	The stem, leaf and seed mixture of *Malva verticillata* L.	[31,35]
**86**	Aspartic acid	C_4_H_7_NO_4_	The fruit of *Malva verticillata* L.	[14,18]
**87**	Threonine	C_4_H_9_NO_3_	The fruit of *Malva verticillata* L.	[14,18]
**88**	Serine	C_3_H_7_NO_3_	The fruit of *Malva verticillata* L.	[14,18]
**89**	Glutamic acid	C_5_H_9_NO_4_	The fruit of *Malva verticillata* L.	[14,18]
**90**	Proline	C_5_H_9_NO_2_	The fruit of *Malva verticillata* L.	[14,18]
**91**	Glycine	C_2_H_5_NO_2_	The fruit of *Malva verticillata* L.	[14,18]
**92**	Valine	C_5_H_11_NO_2_	The fruit of *Malva verticillata* L.	[14,18]
**93**	l-isoleucine	C_6_H_13_NO_2_	The fruit of *Malva verticillata* L.	[14,18]
**94**	Leucine	C_6_H_13_NO_2_	The fruit of *Malva verticillata* L.	[14,18]
**95**	Tyrosine	C_9_H_11_NO_3_	The fruit of *Malva verticillata* L.	[14,18]
**96**	Phenylalanine	C_9_H_11_NO_2_	The fruit of *Malva verticillata* L.	[14,18]
**97**	Histidine	C_6_H_9_N_3_O_2_	The fruit of *Malva verticillata* L.	[14,18]
**98**	Arginine	C_6_H_14_N_4_O_2_	The fruit of *Malva verticillata* L.	[18]

**Table 8:** The other substances identified from *Fructus Malvae*.

No.	Chemical Name	Molecular Formula	Medicinal Parts	Reference
**99**	oleamide	C_18_H_35_NO	The seed of *Malva verticillata* L.	[23]
**100**	1,3-dihydroxyacetone dimer	C_6_H_12_O_6_	The seed of *Malva verticillata* L.	[23]
**101**	5-hydroxymethyl furfural	C_6_H_6_O_3_	The seed of *Malva verticillata* L.	[23]
**102**	2-hydroxy-gamma-butyrolactone	C_4_H_6_O_3_	The seed of *Malva verticillata* L.	[23]
**103**	3,5,6,9-tetrahydroxy-7-megastigmene	C_13_H_24_O_4_	The stem, leaf and seed mixture of *Malva verticillata* L.	[35]

**Table 9:** The research progress on the pharmacological activity of *Fructus Malvae*.

Pharmacological Activity	Compound/Extract	Experimental Level	Experimental Model	Administration Method	Dosage/Concentration	Detection Indicator	Effective Dose	Reference
Diuretic effect	Petroleum ether extract	Whole animal	Water-loaded rat model	Oral administration	25 mL/kg	Urine volume, urine sodium content, urine potassium content, urine chlorine content (mg)	Effective dose: 25 mL/kg	[23]
	Ethyl acetate extract	Whole animal	Water-loaded rat model	Oral administration	25 mL/kg	Urine volume, urine sodium content, urine potassium content, urine chlorine content (mg)	Effective dose: 25 mL/kg	[23]
Anti-diabetic	Ethyl acetate extract	Whole animal	Alloxan-induced islet damage model in zebrafish larvae	Soak absorption	25~600 μg/mL, 10 μg/mL	50% lethal concentration LC50; changes in islet area, changes in fluorescence intensity caused by 2-NBDG	LC_50_:91.5 μg/mL; Effective dose: 10 μg/mL	[18]
	n-Butanol extract	Whole animal	Alloxan-induced islet damage model in zebrafish larvae	Soak absorption	25~600 μg/mL, 10 μg/mL	50% lethal concentration LC50; changes in islet area, changes in fluorescence intensity caused by 2-NBDG	LC_50_:270.9 μg/mL; Effective dose: 10 μg/mL	[18]
		Whole animal	Alloxan-induced islet damage model in zebrafish larvae	Soak absorption	10 μg/mL	Changes in islet area, changes in fluorescence intensity caused by 2-NBDG	Effective dose: 10 μg/mL	[30]
	Water extract	Whole animal	Alloxan-induced islet damage model in zebrafish larvae	Soak absorption	25~600 μg/mL, 10 μg/mL	50% lethal concentration LC50; changes in islet area, changes in fluorescence intensity caused by 2-NBDG	LC_50_:401.1 μg/mL; Effective dose: 10 μg/mL	[18]
	Nortangeretin-8-O-β-d-glucuronide	Whole animal	Alloxan-induced islet damage model in zebrafish larvae	Soak absorption	0.1 μM	Changes in islet area, changes in fluorescence intensity caused by 2-NBDG	Effective dose: 0.1 μM	[18]
	Hypolaetin 8-O-β-d-glucuronopyranoside	Whole animal	Alloxan-induced islet damage model in zebrafish larvae	Soak absorption	0.1 μM	Changes in islet area, changes in fluorescence intensity caused by 2-NBDG	Effective dose: 0.1 μM	[18]
	Herbacetin 8-O-β-d-glucuronopyranoside	Whole animal	Alloxan-induced islet damage model in zebrafish larvae	Soak absorption	0.1 μM	Changes in islet area, changes in fluorescence intensity caused by 2-NBDG	Effective dose: 0.1 μM	[18]
	Isoscutellarein 7-O-β-d-glucopyranoside	Whole animal	Alloxan-induced islet damage model in zebrafish larvae	Soak absorption	0.1 μM	Changes in islet area, changes in fluorescence intensity caused by 2-NBDG	Effective dose: 0.1 μM	[18]
	l-tryptophan	Whole animal	Alloxan-induced islet damage model in zebrafish larvae	Soak absorption	1 μg/m	Changes in islet area, changes in fluorescence intensity caused by 2-NBDG	Effective dose: 1 μg/mL	[30]
	3,5,6,9-tetrahydroxy-7-megastigmene	Whole animal	Alloxan-induced islet damage model in zebrafish larvae	Soak absorption	1 μg/m	Changes in islet area, changes in fluorescence intensity caused by 2-NBDG	Effective dose: 1 μg/mL	[30]
	n-hexane extract	Whole animal	Type 2 diabetes db/db mice	Oral administration	10~40 mg/kg weight/d	Fasting blood glucose levels, non-fasting blood glucose levels, triglycerides, total cholesterol, high-density lipoprotein cholesterol, HTR (high-density lipoprotein cholesterol/total cholesterol), phosphorylation levels of AMPK and ACC in soleus muscle and liver	Effective dose: 20 mg/kg	[32]
	β-sitosterol	Cellular level	L6 myotube cells	Incubation	75~300 μM	Phosphorylation levels of AMPK and ACC, glucose uptake	Effective dose: 75 μM	[32]
	Neutral polysaccharide MVS-Ⅰ	Whole animal	Male mice	Intraperitoneal injection	10~100 mg/kg	0 h, 7 h and 24 h plasma glucose level	Effective dose: 10 mg/kg	[28]
	Peptidoglycan MVS-V	Whole animal	Male mice	Intraperitoneal injection	10~100 mg/kg	0 h, 7 h and 24 h plasma glucose level	Effective dose: 100 mg/kg	[28]
	Peptidoglycan-enriched fraction MVS-V-CH	Whole animal	Male mice	Intraperitoneal injection	10~100 mg/kg	0 h, 7 h and 24 h plasma glucose level	Effective dose: 10 mg/kg	[28]
Anti-oxidation	Nortangeretin-8-O-β-d-glucuronide	Physical and chemical reaction	DPPH RS activity, ABTS RS activity, oxygen-radical absorbance capacity (ORAC) assay, superoxide scavenging activity	Incubation	0.1 mL, 20 μL,	DPPH EC_50_, ABTS EC_50_, ORAC, SOD EC_50_	DPPH EC_50_: >50 µM, ABTS EC_50_: 2.22 ± 0.05 µM, ORAC: 14.38 ± 0.35 µmol TE/µmol SOD EC_50_: 0.73 ± 0.09 µM	[18]
	Isoscutellarein 8-O-β-d-glucuronopyranoside	Physical and chemical reaction	DPPH RS activity, ABTS RS activity, oxygen-radical absorbance capacity (ORAC) assay, superoxide scavenging activity	Incubation		DPPH EC_50_, ABTS EC_50_, ORAC, SOD EC_50_	DPPH EC_50_: >50 µM, ABTS EC_50_: 3.38 ± 0.15 µM, ORAC: 8.06 ± 0.36 µmol TE/µmol, SOD EC_50_:1.51 ± 0.15 µM	[18]
	hypolaetin 8-O-β-d-glucuronopyranoside	Physical and chemical reaction	DPPH RS activity, ABTS RS activity, oxygen-radical absorbance capacity (ORAC) assay, superoxide scavenging activity	Incubation		DPPH EC_50_, ABTS EC_50_, ORAC, SOD EC_50_	DPPH EC_50_: 5.98 ± 0.24 µM, ABTS EC_50_: 1.52 ± 0.04 µM, ORAC: 12.48 ± 1.27 µmol TE/µmol, SOD EC_50_: 0.98 ± 0.13 µM	[18]
	herbacetin 8-O-β-d-glucuronopyranoside	Physical and chemical reaction	DPPH RS activity, ABTS RS activity, oxygen-radical absorbance capacity (ORAC) assay, superoxide scavenging activity	Incubation		DPPH EC_50_, ABTS EC_50_, ORAC, SOD EC_50_	DPPH EC_50_: 31.79 ± 2.22 µM, ABTS EC_50_: 4.51 ± 0.13 µM, ORAC: 6.56 ± 0.32 µmol TE/µmol, SOD EC_50_: 1.04 ± 0.21 µM	[18]
	herbacetin 3-O-β-d-glucopyranosyl-8-O-β-d-glucuronopyranoside	Physical and chemical reaction	DPPH RS activity, ABTS RS activity, Oxygen-radical absorbance capacity (ORAC) assay, superoxide scavenging activity	Incubation		DPPH EC_50_, ABTS EC_50_, ORAC, SOD EC_50_	DPPH EC_50_: 33.80 ± 1.89 µM, ABTS EC_50_: 4.05 ± 0.14 µM, ORAC: 6.42 ± 0.18 µmol TE/µmol, SOD EC_50_: 0.70 ± 0.18 µM	[18]
	isoscutellarein 7-O-d-glucopyranoside	Physical and chemical reaction	DPPH RS activity, ABTS RS activity, oxygen-radical absorbance capacity (ORAC) assay, superoxide scavenging activity	Incubation		DPPH EC_50_, ABTS EC_50_, ORAC, SOD EC_50_	DPPH EC_50_: >50 µM, ABTS EC_50_: 21.62 ± 1.26 µM, ORAC: 3.83 ± 0.30 µmol TE/µmol, SOD EC_50_: 1.31 ± 0.20 µM	[18]
	90% ethanol extract	Physical and chemical reaction	DPPH radical scavenging activity assay	Incubation	100 μL (1–1000 μg/mL)	DPPH anion scavenging activity, ABTS cation scavenging activity, FRAP		[17]
Antitumor	Ethyl acetate extract	Cellular level	Splenocytes, natural killer (NK) cells	Incubation	10 μg/mL	splenocyte proliferation ability, natural killer (NK) cell activity	Effective dose: 10 μg/mL	[24]
		Cellular level	HepG2, AGS, HCT-15, A549	Incubation		50% inhibitory concentration IC50	IC_50_ ± SD: 83.7 ± 3.98 μg/mL, 79.0 ± 1.47 μg/mL, 80.9 ± 1.56 μg/mL, 87.0 ± 0.98 μg/mL,	[33]
	n-butanol extract	Cellular level	Splenocytes, natural killer (NK) cells	Incubation	10 μg/mL	splenocyte proliferation ability, natural killer (NK) cell activity	Effective dose: 10 μg/mL	[24]
		Cellular level	HepG2, AGS, HCT-15, A549	Incubation	10~40 μg/mL	50% inhibitory concentration IC50, AGS cell apoptosis percentage, Expression of apoptosis proteins PARP, Cleaved APRP, Caspase-3, Cleaved Caspase-3, Bcl-2, Bax, β-actin	IC_50_ ± SD: 11.3 ± 0.30 μg/mL, 8.2 ± 0.14 μg/mL, 7.4 ± 0.26 μg/mL, 52.2 ± 4.32 μg/mL,	[33]
	Water extract	Cellular level	Splenocytes, Natural Killer (NK) cells	Incubation	10 μg/mL	splenocyte proliferation ability, natural killer (NK) cell activity	Effective dose: 10 μg/mL	[24]
		Cellular level	HepG2, AGS, HCT-15, A549	Incubation		50% inhibitory concentration IC50	IC_50_ ± SD: 86.0 ± 1.66 μg/mL, 90.0 ± 0.14 μg/mL, 91.5 ± 2.76 μg/mL, 96.3 ± 2.24 μg/mL	[33]
	(2S)-1-O-palmitoyl glyceride	Cellular level	Splenocytes, natural killer (NK) cells	Incubation	10 μM	splenocyte proliferation ability, natural killer (NK) cell activity	Effective dose: 10 μM	[24]
	(2S)-1-O-stearoyl glyceride	Cellular level	Splenocytes, natural killer (NK) cells	Incubation	10 μM	splenocyte proliferation ability, natural killer (NK) cell activity	Effective dose: 10 μM	[24]
	(2S)-1-O-linolenoyl glyceride	Cellular level	Splenocytes, natural killer (NK) cells	Incubation	10 μM	splenocyte proliferation ability, natural killer (NK) cell activity	Effective dose: 10 μM	[24]
	(2S)-1,2-di-O-linoleoyl glyceride	Cellular level	Splenocytes, natural killer (NK) cells	Incubation	10 μM	splenocyte proliferation ability, natural killer (NK) cell activity	Effective dose: 10 μM	[24]
	(2S)-1-O-(6-deoxy-6-sulfo)-α-D glucopyranosyl-2-O-linolenoyl-3-O-palmitoyl glyceride	Cellular level	HepG2, AGS, HCT-15, A549	Incubation	25~100 μM	50% inhibitory concentration IC50, AGS cell apoptosis percentage, Expression of apoptosis proteins PARP, Cleaved APRP, Caspase-3, Cleaved Caspase-3, Bcl-2, Bax, β-actin	IC50 ± SD: 63.7 ± 2.43 μM, 33.7 ± 0.64 μM, 49.6 ± 0.24 μM, 81.8 ± 2.19 μM	[33]
	(2S)-1-O-(6-deoxy-6-sulfo)-α-d-glucopyranosyl-2,3-di-O-linolenoyl glyceride	Cellular level	HepG2, AGS, HCT-15, A549	Incubation	20~80 μM	50% inhibitory concentration IC50, AGS cell apoptosis percentage, Expression of apoptosis proteins PARP, Cleaved APRP, Caspase-3, Cleaved Caspase-3, Bcl-2, Bax, β-actin	IC50 ± SD: 34.7 ± 2.26 μM, 11.1 ± 0.07 μM, 49.2 ± 5.16 μM, 76.0 ± 2.62 μM	[33]
	(2S)-1-O-β-d- galactopyranosyl-3-O-palmitoyl glyceride	Cellular level	HepG2, AGS, HCT-15, A549	Incubation		50% inhibitory concentration IC50	IC50 ± SD: 83.4 ± 0.55 μM, 86.7 ± 2.02 μM, >100 μM, 96.1 ± 2.23 μM	[33]
	(2S)-1-O-β-d-galactopyranosyl-3-O stearoyl glyceride	Cellular level	HepG2, AGS, HCT-15, A549	Incubation		50% inhibitory concentration IC50	IC50 ± SD: 71.1 ± 2.04 μM, 77.7 ± 6.22 μM, >100 μM, 87.5 ± 3.98 μM	[33]
	(2S)-1-O-β-d-galactopyranosyl-3-O-isostearoyl glyceride	Cellular level	HepG2, AGS, HCT-15, A549	Incubation		50% inhibitory concentration IC50	IC50 ± SD: 77.3 ± 1.76 μM, 89.5 ± 0.88 μM, >100 μM, 91.5 ± 1.76 μM	[33]
	(2S)-1-O-β-d-galactopyranosyl-3-O-linolenoyl glyceride	Cellular level	HepG2, AGS, HCT-15, A549	Incubation		50% inhibitory concentration IC50	IC50 ± SD: 74.9 ± 1.89 μM, 89.3 ± 1.21 μM, 91.8 ± 2.43 μM, 89.9 ± 1.61 μM	[33]
	(2S)-1-O-β-d-galactopyranosyl-2,3-di-O-palmitoyl glyceride	Cellular level	HepG2, AGS, HCT-15, A549	Incubation		50% inhibitory concentration IC50	IC50 ± SD: 83.1 ± 0.48 μM, 90.6 ± 1.00 μM, 90.6 ± 1.00 μM, 87.9 ± 2.69 μM	[33]
	(2S)-1-O-β-d-galactopyranosyl-2,3-di-O-linolenoyl glyceride	Cellular level	HepG2, AGS, HCT-15, A549	Incubation		50% inhibitory concentration IC50	IC50 ± SD: 76.3 ± 1.23 μM, 64.8 ± 2.24 μM, 77.5 ± 4.64 μM, >100 μM	[33]
	(2S)-1-O-6′-O-(α-d-galactopyranosyl)-β-d-galactopyra-nosyl-3-O-palmitoyl glyceride	Cellular level	HepG2, AGS, HCT-15, A549	Incubation		50% inhibitory concentration IC50	IC50 ± SD: 74.4 ± 0.78 μM, 70.6 ± 1.00 μM, 85.9 ± 3.33 μM, 87.8 ± 4.53 μM	[33]
	(2S)-1-O-6′-O-(α-d-galactopyranosyl)-β-d-galactopyran-osyl-3-O-linolenoyl glyceride	Cellular level	HepG2, AGS, HCT-15, A549	Incubation		50% inhibitory concentration IC50	IC50 ± SD: 79.3 ± 1.46 μM, 85.4 ± 1.74 μM, 91.3 ± 3.28 μM, 98.3 ± 0.67 μM	[33]
	(2S)-1-O-6′-O-(α-d-galactopyranosyl)-β-d-galactopyrano-syl-2,3-di-O-palmitoyl glyceride	Cellular level	HepG2, AGS, HCT-15, A549	Incubation	10~40 μM	50% inhibitory concentration IC50, AGS cell apoptosis percentage, Expression of apoptosis proteins PARP, Cleaved APRP, Caspase-3, Cleaved Caspase-3, Bcl-2, Bax, β-actin	IC50 ± SD: 10.0 ± 0.45 μM, 10.6 ± 0.10 μM, 15.3 ± 1.12 μM, 7.1 ± 0.12 μM	[33]
	(2S)-1-O-(6-O-α-d-galactopyranosyl)-β-d-galactopyran-osyl-2-O-stearolyl-3-O-linolenoyl glyceride	Cellular level	HepG2, AGS, HCT-15, A549	Incubation		50% inhibitory concentration IC50	IC50 ± SD: 72.8 ± 2.41 μM, 88.2 ± 1.59 μM, 97.1 ± 5.18 μM, >100 μM	[33]
	(2S)-1-O-(6-O-α-d-galactopyranosyl)-β-d-Galactopyrano syl-2-O-stearolyl-3-O-linolenoyl glyceride	Cellular level	HepG2, AGS, HCT-15, A549	Incubation		50% inhibitory concentration IC50	IC50 ± SD: 71.3 ± 0.46 μM, 66.3 ± 1.96 μM, 74.6 ± 1.93 μM, 83.1 ± 3.66 μM	[33]
Hair-loss treatment	95% ethanol extract	Cellular level	HFDPC cells	Incubation	3~100 μg/mL	Cell proliferation rate	Effective dose: 100 μg/mL	[31]
	n-hexane extract	Cellular level	HFDPC cells	Incubation	3~100 μg/mL	Cell proliferation rate	Effective dose: 30 μg/mL	[31]
	Linoleic acid	Cellular level	HFDPC cells	Incubation	3~30 μg/mL	Cell proliferation rate, Wnt/β-catenin signaling pathway proteins GSK-3β, β-catenin; Cyclin D1, CDK2, GAPDH; cell growth factor VEGF, IGF-1, HGF, KGF, GAPDH	Effective dose: 10 μg/mL	[31]
	Ethanol extract	Cellular level	Human dermal papilla cells (DPCs)	Incubation	0~50 μg/mL	Wnt reporter activity, expression of intracellular proteins β-catenin and GAPDH	Effective dose: 10 μg/mL	[21]
	Dichloromethane extract	Cellular level	Human dermal papilla cells DPCs)	Incubation	10~100 μg/mL	Wnt reporter activity, expression of intracellular proteins β-catenin and GAPDH	Effective dose: 10 μg/mL	[21]
	Myristoleic acid	Cellular level	Human dermal papilla cells (DPCs)	Incubation	0~100 μg/mL	Wnt reporter activity, cell number, expression of cytokines IGF-1, KGF, VEGF, HGF, GAPDH, Phosphorylation levels of cell-signaling molecules p-38, ERK, CREB, Akt	Effective dose: 10 μg/mL	[21]

## Data Availability

Not applicable.
